# Massive, Retroperitoneal, Well-Differentiated Liposarcoma in Second-Trimester Pregnancy: A Report of a Rare Case

**DOI:** 10.7759/cureus.63622

**Published:** 2024-07-01

**Authors:** Minh P Nguyen, Phu V Pham, An K Vu, Duy Nguyen, Hung V Tran

**Affiliations:** 1 Department of Gastrointestinal Surgery, Binh Dan Hospital, Ho Chi Minh, VNM; 2 Department of General Surgery, Pham Ngoc Thach Medical University, Ho Chi Minh City, VNM

**Keywords:** magnetic resonance image, well-differentiated liposarcoma, second trimester, retroperitoneal, liposarcoma

## Abstract

Retroperitoneal liposarcoma during pregnancy is rare and poses significant diagnostic challenges, even for experienced specialists. We present a case report of a 27-year-old female patient, 15 weeks pregnant, who was admitted to the hospital due to a massive retroperitoneal liposarcoma. The patient underwent surgical resection of the tumor. Postoperative pathology confirmed a diagnosis of well-differentiated liposarcoma. Although liposarcoma during pregnancy is rare and challenging to diagnose, CT or MRI plays a crucial role in its detection. The recurrence rate depends on the pathological stage, histological grade, and ability to resect the tumor.

## Introduction

Retroperitoneal liposarcoma in pregnancy is extremely rare and often causes difficulties in diagnosis, even for specialists. Symptoms are often nonspecific and can easily be mistaken for those related to pregnancy [1]. This type of tumor can occur at any age but is most common between the ages of 40 and 70 [2,3]. In the world literature, only a few cases of retroperitoneal liposarcoma during pregnancy have been reported, and it is not clear whether there is any link between this tumor and pregnancy [4]. According to the World Health Organization, liposarcoma is classified into five types based on histopathology: well-differentiated, myxoid, round cell, pleomorphic, and de-differentiated [5].

Radical resection of the tumor is the standard treatment for this disease. However, an individualized approach for each patient is essential. Patient age, underlying medical condition, gestational age, tumor location, and fetal status should be considered before surgical resection [6].

Here, we present a case of a 15-week pregnant patient with a massive retroperitoneal liposarcoma treated by complete resection.

## Case presentation

A 27-year-old female patient was admitted to the hospital with a complaint of vague, intermittent abdominal pain for the last two years and increased intensity over the last three months. She was in her fifteenth week of gestation at the time of presentation. This patient only visited the local medical center once during the antenatal care period in the suburb with a pregnancy confirmation. No pre-existing medical conditions were noted. Clinical examinations were within normal range. The patient's abdomen was abnormally large for gestational age. There was an intra-abdominal mass of about 30x40 cm with indistinct margins.

**Figure 1:**
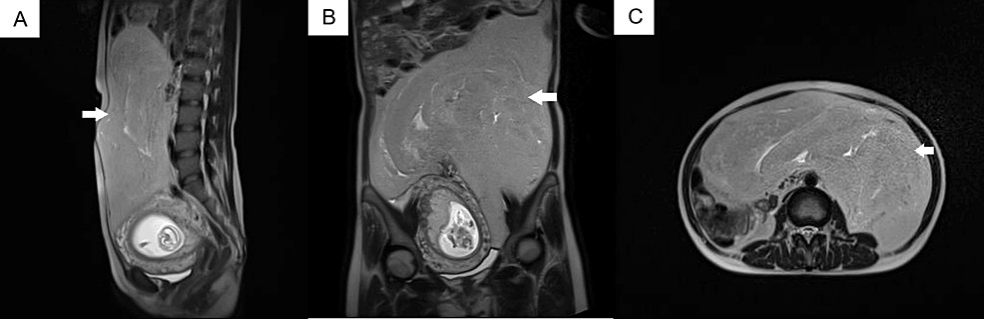
Axial (A), coronal (B), and sagittal (C) magnetic resonance imaging (MRI) images of massive retroperitoneal liposarcoma

Blood tests were within normal limits. The MRI image showed a massive thick-septa tumor of 30x30x20x40 cm, resembling adipose tissue in density with high signal intensity on T1WI and T2WI and a low but heterogeneous intensity signal on Fat-Sat pulses (Figure 1). The tumor originated retroperitoneally. Liver metastasis, urinary system, and major vessel involvement were not recorded. Because of its large size, the tumor abutted surrounding organs and directly compressed the uterus, causing danger to the fetus during development. Surgical tumor resection was planned due to symptoms and to minimize risk to the fetus.

**Figure 2:**
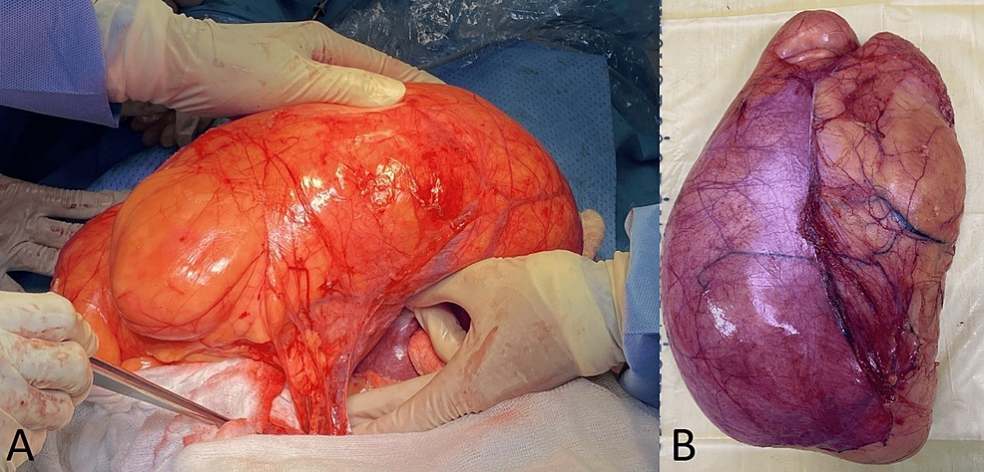
Massive liposarcoma with a bundle of blood vessels (A); the massive liposarcoma taken outside (B)

The retroperitoneal location of the tumor was confirmed during exploration, with a sizeable vascular bundle supplying blood that originated from the right ovarian vessels (Figure 2A). The tumor was abutting surrounding organs without infiltrating. The uterus was displaced to the left. The general surgeons removed the tumor entirely and did not cause damage to nearby organs. The tumor mass was 5 kg (Figure 2B).

**Figure 3:**
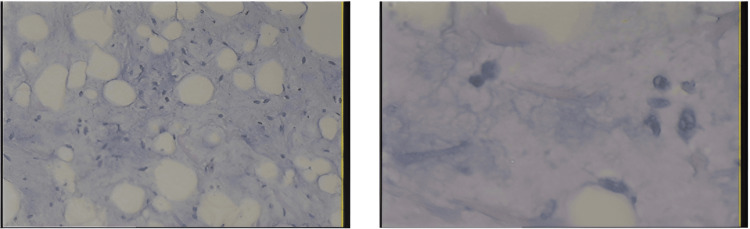
Microscopic appearance of a well-differentiated primary retroperitoneal liposarcoma

Postoperatively, no complications were noted. Obstetric examination revealed a healthy fetus. The patient was discharged on the fifth postoperative day and was re-examined one week later without any abnormalities. Postoperative pathology revealed well-differentiated liposarcoma (Figure 3). Re-examination after one month did not record any abnormal symptoms or recurrence signs on ultrasonography. The patient then had an uneventful pregnancy course with a vaginal delivery three months after the surgery. A CT scan was planned for a local recurrence check. However, we lost contact with the patient since postpartum.

## Discussion

Among soft tissue sarcomas, liposarcoma is the most common. This pathology is shared between the ages of 40 and 70. Liposarcoma can occur in many places in the body, but the most common locations are in the extremities and retroperitoneum [7]. Depending on the location, the impact of liposarcoma on the body is different.

The overall survival rate is between 23% and 46%. The ability to radically resect the tumor and histological grade are patients’ most important prognostic factors [8,9]. Well-differentiated liposarcoma is the most common histological group. This type of tumor usually does not cause invasion or distant metastases and, therefore, has the best prognosis of the five types [3]. The survival and prognosis of liposarcoma are not affected by pregnancy [4]. Metastasis in the fetus or placenta is extremely rare [10].

Liposarcoma in pregnancy is rare and, therefore, difficult to diagnose. In most cases, there are no apparent symptoms, and they are often confused with fetal development. The nature of the tumor's manifestation significantly affects patients who are far away from medical facilities and unable to have regular antenatal visits. In other cases, abdominal or pelvic pain is the reason for the patient's visit [11]. At a later stage, when the tumor is large and compresses nearby organs, symptoms may appear more often. Localized pain may occur when the tumor compresses a particular nerve plexus, and gastric compression can cause vomiting or upper gastrointestinal bleeding. Large tumors can also cause secondary hypertension, constipation, diarrhea, cramping abdominal pain, hydronephrosis, and pyelonephritis [8]. Diagnosis is, therefore, often made accidentally during antenatal care. CT or MRI has the most critical role in the diagnosis [9]. Currently, radical surgical resection of liposarcoma must be performed to achieve a high therapeutic effect [12]. Achieving a negative surgical margin during surgery may improve prognosis, with 5-year overall survival rates ranging from 68% to 80%. However, local recurrence rates can still be as high as 75% [3,13].

In our case, the patient did not have access to appropriate antenatal care during the first trimester, which led to a late diagnosis in the second trimester when her abdomen was abnormally enlarged and abdominal pain increased in intensity. Vague abdominal pain has been prolonged for two years. However, pregnancy with a growing fetus significantly contributed to increasing compression on surrounding structures, which intensified the pain during the first trimester. The patient was young and willing to preserve the fetus; the diagnosis was made in her second trimester, a time associated with a reduced risk of spontaneous abortion, which was suitable for performing a surgical resection to remove the tumor. The patient experienced an uneventful postoperation period and successfully delivered three months later. However, follow-up re-examination was not applicable as we lost contact with the patient.

## Conclusions

We reported a rare case of giant retroperitoneal liposarcoma during the second trimester of pregnancy. Giant retroperitoneal liposarcoma is extremely rare, with nonspecific symptoms, which can pose a challenge for physicians to recognize and properly manage both the tumor and the fetus. The primary treatment is to remove the tumor with a safety margin, if possible, excluding the area related to vital structures. The recurrence rate depends on the pathological stage, histological grade, and negative surgical margin. The preservation of the fetus is feasible with total resection of the tumor. Lifetime follow-up is indicated, as the recurrence rate cannot be ignored in the case of liposarcoma.
